# Tail spillover effects between cryptocurrencies and uncertainty in the gold, oil, and stock markets

**DOI:** 10.1186/s40854-023-00498-y

**Published:** 2023-05-03

**Authors:** Walid Mensi, Mariya Gubareva, Hee-Un Ko, Xuan Vinh Vo, Sang Hoon Kang

**Affiliations:** 1 Department of Economics and Finance, College of Economics and Political Science, Sultan Qaboos University, Muscat, Oman; 2 Institute of Business Research, University of Economics Ho Chi Minh City, Ho Chi Minh City, Vietnam; 3 Universidade de Lisboa, Lisbon School of Economics and Management (ISEG), Research Centre in Economic and Organisational Sociology (SOCIUS) / Research in Social Sciences and Management (CSG), Rua Miguel Lupi 20, 1249-078 Lisbon, Portugal; 4 Korea Housing and Urban Guarantee Corporation, Busan, Korea; 5 Institute of Business Research and CFVG, University of Economics Ho Chi Minh City, Ho Chi Minh City, Vietnam; 6 PNU Business School, Pusan National University, Busan, Republic of Korea

**Keywords:** Cryptocurrency, Uncertainty indices, Quantile spillover, Cross-quantilogram, G14, F36, C40

## Abstract

This study investigates tail dependence among five major cryptocurrencies, namely Bitcoin, Ethereum, Litecoin, Ripple, and Bitcoin Cash, and uncertainties in the gold, oil, and equity markets. Using the cross-quantilogram method and quantile connectedness approach, we identify cross-quantile interdependence between the analyzed variables. Our results show that the spillover between cryptocurrencies and volatility indices for the major traditional markets varies substantially across quantiles, implying that diversification benefits for these assets may differ widely across normal and extreme market conditions. Under normal market conditions, the total connectedness index is moderate and falls below the elevated values observed under bearish and bullish market conditions. Moreover, we show that under all market conditions, cryptocurrencies have a leadership influence over the volatility indices. Our results have important policy implications for enhancing financial stability and deliver valuable insights for deploying volatility-based financial instruments that can potentially provide cryptocurrency investors with suitable hedges, as we show that cryptocurrency and volatility markets are insignificantly (weakly) connected under normal (extreme) market conditions.

## Introduction

In recent years, the issues of cryptocurrency market contagion, market uncertainty, and market complexity, combined with elevated volatility in major traditional markets, have attracted much interest from academic researchers and market practitioners (Antonakakis et al. 2019; Xu et al. 2019; Umar and Gubareva 2020; Bouri et al. 2021; Sebastião and Godinho 2021; Fang et al. 2022; Ghorbel et al. 2022; Maghyereh and Abdoh 2022; Mandaci and Cagli 2022; Ren and Lucey 2022; Salisu and Ogbonna 2022; Umar et al. 2022a; Bossman and Gubareva 2023; Yousaf et al. 2023). One prominent area of research in this field centers on the risk spillover between cryptocurrencies and traditional financial markets, with a particular focus on new avenues for hedging and diversification opportunities in both directions (Hsu et al. 2021; Zhang and He 2021; Attarzadeh and Balcilar 2022; Maitra et al. 2022; Yousaf et al. 2022; Bossman et al. 2023a,b).

Several studies have investigated quantile connectedness, risk diffusion, contagion, and tail spillover in crypto-markets (Bouri et al. 2021; Jena et al. 2020; Mensi et al. 2021b; Xu et al. 2021; Naeem et al. 2022; Yang et al. 2022) and commodity markets (Umar et al. 2021b, c; Umar et al. 2022c; Hanif et al. 2023). In parallel, both connectedness and elevated uncertainty in traditional financial markets have been widely studied recently (Bams et al. 2017; Godil et al. 2020; Roh et al. 2020; Zhang et al. 2021; Benlagha and El Omari 2022; Mensi et al. 2022). However, to the best of our knowledge, no research to date has examined the relationships between cryptocurrencies and uncertainty in traditional markets. Our study aims to bridge this gap by providing empirical evidence on the leading role in the spillover of the most prominent cryptocurrencies vis-à-vis the volatility dynamics of the equity, oil, and gold markets.

Our study adds to the existing literature by analyzing upside and downside spillover effects between five major cryptocurrencies and uncertainty (volatility) indices for major traditional financial assets. Our motivation in studying these markets in this way is directly linked to their importance and popularity among academic researchers and practitioners, as evidenced by the high quality research covering crypto-assets and conventional financial instruments. Our research employs the advanced cross-quantilogram method (Han et al. 2016) and quantile connectedness approach (Ando et al. 2022) to identify cross-quantile interdependencies between the variables analyzed and investigate tail spillover between the crypto- and traditional markets in their lower, middle, and upper return distributions. Using these methodologies we show that the spillover between cryptocurrencies and major conventional market volatility indices varies substantially across quantiles, implying that their diversification benefits may differ widely across normal and extreme market conditions. We document the relationships between variables, analyze them using the connectedness framework and find new hedging and diversification opportunities in portfolio management.

This study aims to examine the dependence within and between main cryptocurrencies and uncertainty indices of stock, oil, and gold under bearish, tranquil, and bullish market states. We also assess the dynamic spillover size and direction, as well as connectedness in different cryptocurrency market scenarios.

Our results show that the spillover between cryptocurrencies and volatility indices for traditional markets varies substantially across quantiles, implying that the diversification benefits of these assets may differ widely across normal and extreme market conditions. We find that the total connectedness index (TCI) under normal market conditions is moderate and lower than the elevated values observed for bearish and bullish markets. Furthermore, we show that, regardless of the market state, the cryptocurrencies Bitcoin (BTC), Litecoin (LTC), Ethereum (ETH), and Bitcoin Cash (BCH) always behave as net pairwise transmitters of shocks to Ripple (XRP) and the volatility indices. In addition, cryptocurrencies exercise leadership influence over the volatility indices in all market states. Our results have important implications regarding the use of volatility-based financial instruments, which can potentially provide suitable hedges for crypto-investors, as we show that crypto- and volatility markets are insignificantly (weakly) connected under normal (extreme) market conditions.

Our study contributes to the existing literature on different fronts. First, it examines the quantile dependence between leading cryptocurrencies and the volatility indices of equity, oil, and gold markets, advancing the current knowledge of the main strands of research in the field. It contributes to the literature on tail risk (i.e., the risk of rare or extreme events), which is among the most relevant risks for investment and portfolio management (Kelly and Jiang 2014; Fendel and Neumann 2021; Mensi et al. 2021b). Identifying the sources of tail risk is essential to understanding the impact of that risk on portfolio performance, especially as exposure to tail risk may crystallize the crash risk of investment portfolios and decrease overall returns on investments (Agarwal et al. 2017). Therefore, the research community remains acutely interested in assessing and managing tail risk for both traditional financial instruments (Happersberger et al. 2020; Mensi et al. 2021b) and emergent digital assets such as cryptocurrencies, nonfungible tokens (NFTs), and decentralized finance (DeFi). (Borri 2019; Umar and Gubareva 2020; Hsu et al. 2021; Xu et al. 2021; Ando et al. 2022; Naeem et al. 2022; Umar et al. 2022a; Yousaf et al. 2022). our investigation into the tail spillover between cryptocurrencies and uncertainty in the major traditional markets helps to extend this literature stream by providing a better understanding of diversification opportunities and hedging possibilities that can be captured from joint exposure to crypto-assets and traditional stock, oil, and gold markets. This is potentially useful to financial practitioners in designing and managing investment portfolios that incorporate cryptocurrency exposure to increase returns while diversifying nonidiosyncratic risks. Information on tail spillover across distinct markets can also assist policymakers in improving and maintaining financial stability.

Second, we use the cross-quantilogram method in Han et al. (2016) to gauge the interdependence among diverse assets. Following this methodology, we quantitatively assess the interdependence between assets across a broad spectrum of quantiles by comparing the magnitude of influence in the lower, middle, and upper parts of their distributions (i.e., in the center and at the tails). This allows us to identify asymmetric interrelationships among assets under bearish, normal, and bullish market conditions, characterized by extremely low, moderate, and extremely high returns, respectively. This is especially the case for the period from May 4, 2018 to May 7, 2022 which includes the COVID-19 pandemic as well as Russia’s invasion of Ukraine.

To gauge the cross-quantile interdependence between cryptocurrencies and volatilities in the equity, oil, and gold markets, we use five cryptocurrencies—BTC, ETH, LTC, XRP, and BCH—and three CBOE volatility indices that measure equity market volatility (VIX), crude oil volatility (OVX), and gold volatility (GVZ). Our choice of these volatility indices is justified by the fact that they capture volatility observed in three major traditional financial markets. These indices represent investors’ uncertainty regarding the future level of these markets and reflect turbulence in macroeconomic factors, commodity prices, and the stock market (Bams et al. 2017). Interestingly, equity and oil markets respond to various market shocks, while gold is less vulnerable to such shocks as it is commonly accepted as a safe-haven asset in times of crisis (Phan et al. 2018; Hoang et al. 2015). Therefore, these indices contain useful information for investors to use in predicting future price levels and identifying periods of market stability and turbulence. This, in turn, is helpful for portfolio risk management and fund allocation. The selected cryptocurrencies are characterized by their relatively large market capitalizations and trading volumes; BTC and ETH cryptocurrencies together account for over half the value of the global cryptocurrency market.^1^ While ETH and LTC prices have surged several thousand percent since early 2017 and have experienced a huge jump in trading volume (Bouri et al. 2019), we note that cryptocurrencies are far less liquid than traditional security asset classes (Bianchi et al. 2022).

We employ the cross-quantilogram method and the quantile connectedness approach to investigate the tail dependence between the analyzed variables and downside and upside spillover among the markets represented by the chosen volatility indices. The cross-quantilogram method provides some advantages over other methodologies that could be used to gauge the tail interdependence between assets, such as copulas (Tachibana 2018; Xia et al. 2019), (Naeem et al. 2021, 2022; Ando et al. 2022). Specifically, copula-based methodologies would require us to select an appropriate marginal distribution (Vuuren and de Jong 2017); however, instead of requiring an inherently subjective choice of a distribution, the cross-quantilogram method relies on objectively determined quantiles, without depending on any assumptions or conditions regarding the moments of the distribution. Additionally, in contrast to copula-based methodologies, the cross-quantilogram method allows us to analyze as many quantiles and lags as necessary, providing a sufficiently refined view of the magnitude, direction, and elapsed time of the interdependence among variables for a broad spectrum of quantiles (here, the lower extreme, middle and upper extreme portions of the distribution). Hence, the cross-quantilogram method is more suitable than a copula approach for describing the interrelationships among asset returns and investigating tail downside and upside spillover effects (Han et al. 2016; Adekoya et al. 2021; Bouri et al. 2021; Ando et al. 2022; Khalfaoui et al. 2022; Naeem et al. 2022).

The remainder of this study is organized as follows. Sect. "Data and preliminary analysis" presents the data and preliminary analysis. Sect. "Empirical methods" presents the methodology, Sect. "Empirical results" discusses the results, and Sect. "Conclusions" concludes.

## Data and preliminary analysis

**Fig. 1:**
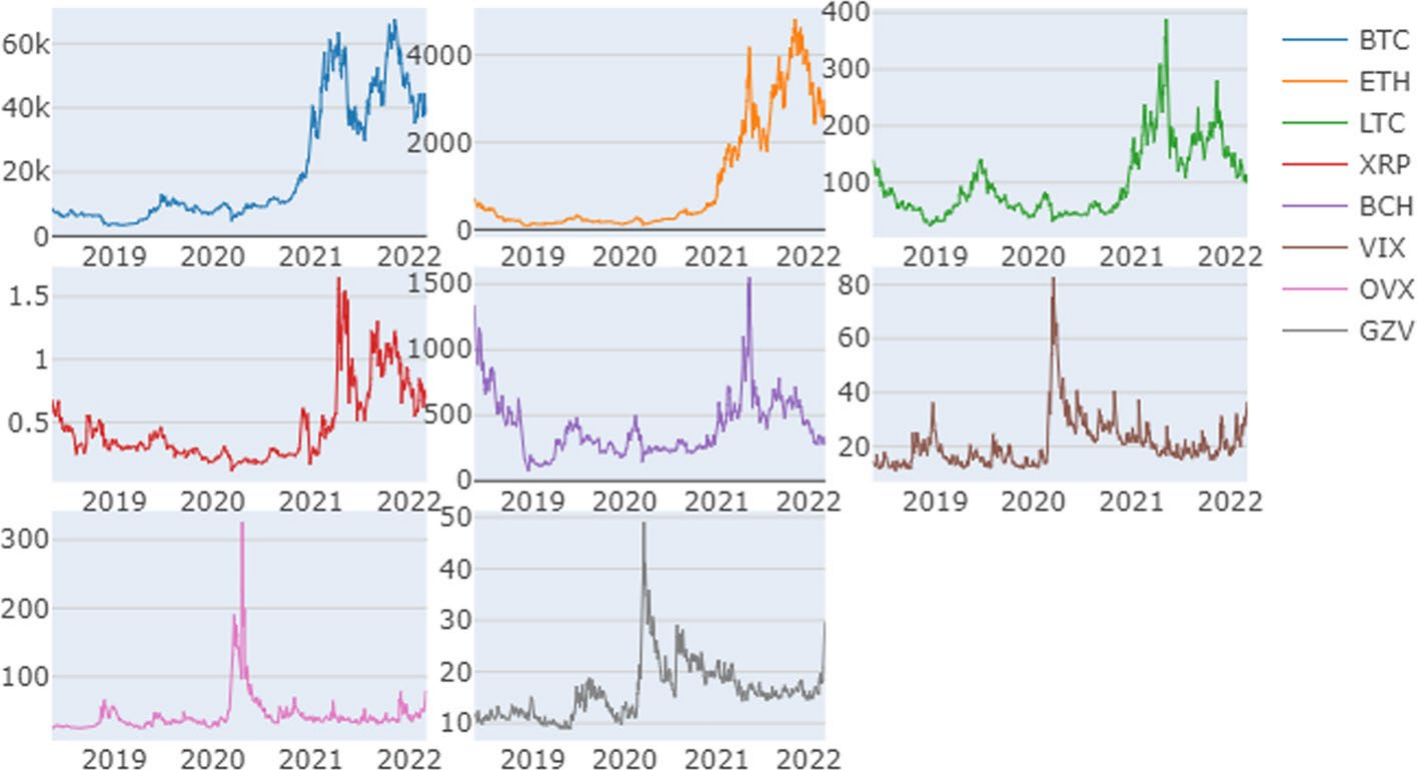
Time-variations in cryptocurrency prices and VIX, OVX, and GZV volatility indices

As noted above, we consider five cryptocurrencies (BTC, ETH, LTC, XRP, and BCH), and three uncertainty indices (i.e., VIX, OVX, and GVZ) listed on the Cboe. Our data sample covers the period from May 4, 2018 to May 7, 2022. We calculate continuously compounded daily returns by taking the difference between the log values of two consecutive prices. Figure 1 displays the evolution of cryptocurrency market prices along with the time dynamics of the chosen volatility indices. We observe significant spikes in the volatility metrics in late March 2020, and a considerable decrease in the value of cryptocurrencies. We ascribe this to the extreme level of uncertainty in global financial markets caused by the uncontrolled spread of COVID-19 in the first quarter of 2020 that triggered an immediate economic slowdown and heightened risk aversion, accompanied by sell-offs in many asset classes, including digital currencies.

**Fig. 2:**
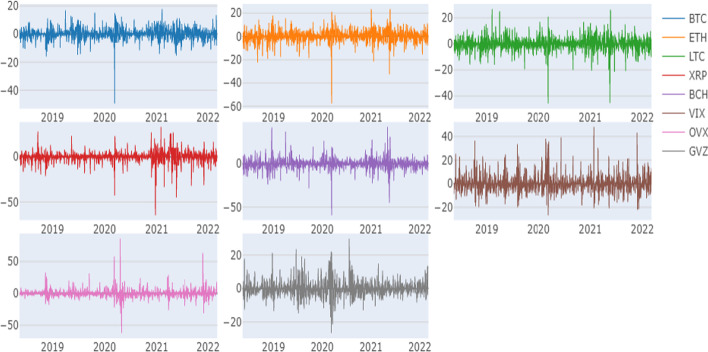
Time-variations in cryptocurrency price returns and VIX, OVX, and GZV volatility indices

Figure 2 presents the time dynamics of the returns of the cryptocurrencies and volatility indices. We observe pronounced negative spikes in cryptocurrency returns near the COVID-19 crisis apogee in late March 2020, corresponding to the augmented volatility of returns of the uncertainty indices. Moreover, all charts show volatility clustering, suggesting nonlinear behavior in all return series.

Table 1 reports basic statistics for the price return series of the various cryptocurrencies and volatility indices. Mean returns are positive for all but LTC and BCH. BTC (0.1077) and ETH (0.0904) exhibit the highest and second highest mean returns, respectively, while the XRP’s mean return, although positive, is close to zero. Among cryptocurrencies, BCH exhibits the largest swings as demonstrated by its maximum and minimum values, closely followed by XRP. Their standard deviations also indicate that these two crypto-assets are highly volatile. We also observe that returns for all of the cryptocurrencies are negatively skewed, whereas the volatility indices exhibit positive skewness. This may be due to the different nature of the time series, and by the fact that as an asset class, volatility is more difficult to trade than cryptocurrencies. Nevertheless, all the return series are asymmetric (based on skewness) and leptokurtic (shown by kurtosis values). In line with these preliminary findings, the normal distribution hypothesis is rejected by the Jarque–Bera test at the 1% significance level for all return series. Both the ADF and KPSS statistics show that all of the return series are stationary.

**Table 1:** Descriptive statistics of cryptocurrency price returns and uncertainty indices

	BTC	ETH	LTC	XRP	BCH	VIX	OVX	GVZ
Mean (%)	0.1077	0.0904	− 0.0250	0.0024	− 0.1137	0.0655	0.0655	0.0780
Max	17.77	23.40	26.69	32.98	42.54	48.02	29.76	85.77
Min	− 49.39	− 57.56	− 45.74	− 64.52	− 59.37	− 26.62	− 26.56	− 62.22
Std. Dev	3.9631	5.2249	5.4344	6.1668	6.1876	7.0185	4.4035	6.6951
Skewness	− 1.3435	− 1.2058	− 0.7503	− 1.6072	− 0.2649	1.3683	0.7883	2.3693
Kurtosis	19.06	13.08	8.839	16.59	13.75	6.970	6.267	36.68
Jarque–Bera	21,507***	10,269***	4666.2***	16,587***	11,000***	3255.1***	2424.1***	79,392***
Q (20)	33.74***	57.73***	36.41***	22.47	35.13***	32.44***	41.02***	35.74***
ADF	− 21.49***	− 21.03***	− 21.71***	− 22.46***	− 20.48***	− 22.91***	− 22.71***	− 23.79***
KPSS	0.1905	0.5459	0.1486	0.1247	0.1674	0.0250	0.0505	0.0327

This table reports the descriptive statistics of sample returns, the Ljung–Box test (Q(20)) for the autocorrelation of returns series, ADF unit root test of Dickey–Fuller (1979), and KPSS stationary test of the Kwiatkowski et al. (1992). The asterisk *** stands for significance at the 1% level

**Fig. 3:**
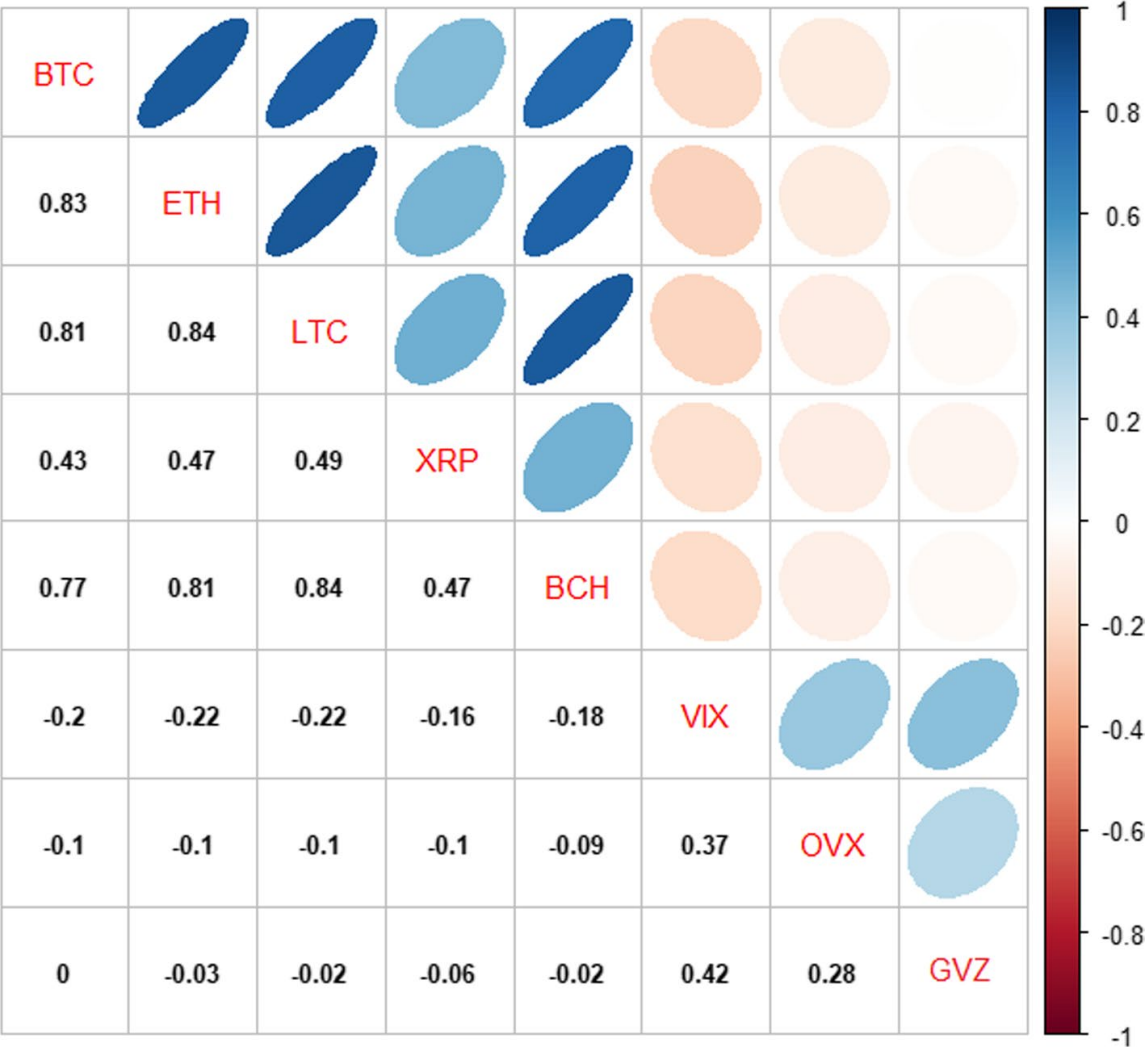
Unconditional correlations between cryptocurrencies and volatility indices. *Notes*: The colored disks indicate the magnitude of the correlation. The color represents the sign of a pairwise correlation (blue = positive and red = negative)

Figure 3 shows the unconditional pairwise correlation matrix for the five cryptocurrencies and three volatility indices. BTC, ETH, LTC, and BCH represent the most correlated clusters, with pairwise correlation coefficients of approximately 0.80, while XRP shows rather weak pairwise correlations with the other cryptocurrencies, with coefficients below 0.50. However, all of these cryptocurrencies are positively correlated with each other, offering limited space for diversification. These results are consistent with the findings in Umar and Gubareva (2020) and Lesame et al. (2021). However, the returns for the three volatility indices, although weakly correlated, are negatively correlated with the returns of the five crypto-markets. Notably, the VIX index shows the most negative correlation coefficients with the cryptocurrencies. The slightly negative pairwise correlation coefficients between the OVX and GVZ volatility indices and the cryptocurrencies, are near zero, showing that these two indices are uncorrelated with the crypto-markets and thus may offer appealing hedge opportunities for portfolios that hold cryptocurrencies based on the underlying commodities linked to the OVX and GVZ indices.

## Empirical methods

### The cross-quantilogram method

We use the cross-quantilogram (CQ) method in Han et al. (2016), which measures the extreme dependence between cryptocurrencies at different quantiles. The CQ method can incorporate the heavy tail features of time series and various time lags and quantifies the strength of dependence across investment horizons (short-term, medium-term, long-term) and market conditions (bearish, normal, and bullish market).

Let ${y}_{i.t}$ be stationary time series, where $i$ represents daily returns for cryptocurrencies $\left(i=1, 2;t=1, \ldots , T\right)$,${F}_{i}\left(\cdot \right)$ represents the cumulative distribution function, and ${f}_{i}\left(\cdot \right)$ the cumulative density function of series ${y}_{i,t}$. The conditional distribution function and quantiles of distributions ${y}_{i,t}$ can be represented as ${F}_{{y}_{i}|{x}_{i}}(\cdot |{x}_{it})$ and ${q}_{i}\left({\alpha }_{i}\right)=inf\left\{v:{F}_{i}(v)\ge {\tau }_{i}\right\}$ for ${\tau }_{i}\upepsilon \left[\mathrm{0,1}\right]$, for $i=1, 2$. Suppose that $\mathrm{\alpha }$ denotes the range of quantiles. Then, the CQ method captures the serial dependence between two series such as $\left\{{y}_{1,t}\le {q}_{1,t}\left({\tau }_{1}\right)\right\}$ and $\left\{{y}_{2,t-k}\le {q}_{2. t-k}\left({\tau }_{2}\right)\right\}$, where the right-hand sides of these inequalities are quantile levels. The CQ method is specified as the cross-correlation of the quantile-hit process of $\alpha$-quantile and $k$-lag given as:

1 $${\rho }_{\tau }(k)=\frac{E\left[{\Psi }_{{\tau }_{1}}({y}_{1,t}- {q}_{1,t}{(\tau }_{1})){\Psi }_{{\tau }_{2}}({y}_{2,t-k}- {q}_{2,t-k}({\tau }_{2}))\right]}{\sqrt{E\left[{\Psi }_{{\tau }_{1}}^{2}({y}_{1,t}- {q}_{1,t}{(\tau }_{1}))\right]}\sqrt{E\left[{\Psi }_{{\tau }_{2}}^{2}({y}_{2,t-k}- {q}_{2,t-k}{(\tau }_{2}))\right]}},$$

where $k$ is the number of lags to time $t$ and ${\Psi }_{\tau }(\mu )$ ≡,$\left[\upmu <0\right]$; 1 $\left[{y}_{i,t}\le {q}_{i,t}{(\tau }_{i})\right]$ represents the quantile hit or exceedance process, where 1 $\left[.\right]$ Is an indicator function. In Eq. (1), the quantile-hit process is determined under time $t-k$, and ${\rho }_{t-k}$ is the correlation of the quantile-hit process.

An unconditional cross-quantile case can be defined as follows: ${\widehat{\uprho }}_{\uptau }\left(\mathrm{k}\right)= \frac{{\sum }_{\mathrm{t}=\mathrm{k}+1}^{\mathrm{T}}{\Psi }_{{\tau }_{1}}({\mathrm{y}}_{1,\mathrm{t}}-{\mathrm{q}}_{1}\left({\uptau }_{1}\right)){\Psi }_{{\tau }_{2}}({\mathrm{y}}_{2,\mathrm{ t}-\mathrm{k}}-{\widehat{\mathrm{q}}}_{2,\mathrm{t}-\mathrm{k}}\left({\uptau }_{2}\right))}{\sqrt{{\sum }_{\mathrm{t}=\mathrm{k}+1}^{\mathrm{T}}{\Psi }_{{\tau }_{1}}^{2}({\mathrm{y}}_{1,\mathrm{ t}}-{\widehat{\mathrm{q}}}_{1,\mathrm{ t}-\mathrm{k}}\left({\uptau }_{1}\right))}\sqrt{{\sum }_{\mathrm{t}=\mathrm{k}+1}^{\mathrm{T}}{\Psi }_{{\tau }_{2}}^{2}({\mathrm{y}}_{2,\mathrm{ t}-\mathrm{k}}-{\widehat{\mathrm{q}}}_{2,\mathrm{t}-\mathrm{k}}\left({\uptau }_{2}\right))}}$*,* (2)

where ${\widehat{q}}_{i}\left({\tau }_{i}\right)$ is an unconditional sample counterpart of a ${q}_{i}\left({\tau }_{i}\right)$ return series ${\mathrm{y}}_{\mathrm{i},\mathrm{t}}$. To test the null hypothesis ${H}_{0}: {\rho }_{\tau }\left(k\right)=0$ against the alternative hypothesis ${H}_{1}: {\rho }_{\tau }\left(k\right)\ne 0$, we use the quantile version of the Ljung–Box–Pierce statistic:

3 $${\widehat{\mathrm{Q}}}_{\uptau }^{(\mathrm{p})}=\frac{\mathrm{T}\left(\mathrm{T}+2\right)\sum_{\mathrm{k}=1}^{\mathrm{p}}{\widehat{\uprho }}_{\uptau }^{2}(\mathrm{k})}{\mathrm{T}-\mathrm{k}},$$

where ${\widehat{\mathrm{Q}}}_{\uptau }^{(\mathrm{p})}$ is a portmanteau-type statistic that tests for the presence of directional predictability or serial dependence between BTC and other cryptocurrencies. We conduct the test for lag orders 1, 5, 22, and 66, corresponding to the directional predictability for daily, weekly, monthly, and quarterly horizons, respectively. To estimate the null distribution of the cross-quantilograms and the Q-statistic, we use the stationary bootstrap (SB) procedure (Politis and Romano 1994). The SB approach is a block bootstrap method using blocks of random lengths. This approach provides the underlying autocorrelation structure and strictly stationary resampling data.

To control for the effect of the trends of the volatility indices (VIX, OVX, and GVZ) on the cross-quantile relationship between BTC and other cryptocurrencies, we apply the partial cross-quantilogram (PCQ) model in Han et al. (2016), incorporating control variables as intermediate events between $t$ and $t - k$*.* The PCQ model includes control variables in the model represented by the vector ${z}_{t}\equiv {\left[{\psi }_{{\tau }_{3}}\left({y}_{3t}-{q}_{3,t}\left({\tau }_{3}\right)\right),\dots ,{\psi }_{{\tau }_{l}}\left({y}_{lt}-{q}_{l,t}\left({\tau }_{l}\right)\right)\right]}^{\intercal }$ where $l=3,\ldots ,n$, and an $\left(l-2\right)\times 1$ vector for $l\ge 3$ control variables. The correlation matrix of the hit processes and their corresponding inverse matrix are presented as:

4 $${R}_{\overline{\tau }}^{-1}=E{\left[{h}_{t}\left(\overline{\tau }\right)h{\left(\overline{\tau }\right)}^{\intercal }\right]}^{-1}={P}_{\tau },$$

where ${h}_{t}\left(\overline{\tau }\right)={\left[{\psi }_{{\tau }_{1}}\left({y}_{1t}-{q}_{1,t}\left({\tau }_{1}\right)\right),\dots ,{\psi }_{\tau l}\left({y}_{lt}-{q}_{l,t}\left({\tau }_{l}\right)\right)\right]}^{\intercal }$ is an $l\times 1$ vector of the quantile hit process and ${P}_{\tau }$ is the PCQ model, defined as:

5 $${{\rho }_{\overline{\tau }|z}=-p}_{\overline{\tau },12}/\sqrt{{p}_{\overline{\tau },11}{p}_{\overline{\tau },22}},$$

where the cross-quantilogram dependence ${\rho }_{\overline{r }|z}$ is conditional on the control variable $z$. Alternatively, ${\rho }_{\overline{\tau }|z}$ can be expressed as

6 $${\uprho }_{\overline{\uptau }|\mathrm{z}}=\updelta \sqrt{\frac{{\uptau }_{1}(1-{\uptau }_{1})}{{\uptau }_{2}(1-{\uptau }_{2}}},$$

where $\updelta$ is a scalar parameter derived from the following regression:

7 $${\uppsi }_{{\tau }_{1}}\left({\mathrm{y}}_{1\mathrm{t}}-{\mathrm{q}}_{1,\mathrm{t}}\left({\uptau }_{1}\right)\right)=\updelta {\uppsi }_{\uptau 2}\left({\mathrm{y}}_{2\mathrm{t}}-{\mathrm{q}}_{2,\mathrm{t}}\left({\uptau }_{2}\right)\right)+{\upgamma }^{\intercal }{\mathrm{z}}_{\mathrm{t}}+{\mathrm{u}}_{\mathrm{t}}.$$

Using the PCQ model, we test the null hypothesis ${\uprho }_{\overline{\uptau }|\mathrm{z}}=0$ against the alternative ${\uprho }_{\overline{\uptau }|\mathrm{z}}\ne 0$. Hence, we measure the serial dependence and directional predictability between the quantile hits of two variables, assuming that quantile hits depend on the information set embedded in the vector ${z}_{t}$.

### Quantile connectedness approach

Following Ando et al. (2022), we apply the quantile connectedness approach to calculate spillover indices in various quantiles $\left(\tau \right)$ based on a quantile variance decomposition. Using this approach we measure the dynamics of connectedness during bearish, normal, and bullish market conditions.

Let us define an infinite-order vector moving average representation of a quantile vector autoregression QVAR(p) as follows:

8 $${y}_{t}=\mu \left(\tau \right)+{\sum }_{j}^{p}{\Phi }_{j}\left(\tau \right){y}_{t-j}+{u}_{t}\left(\tau \right)=\mu \left(\tau \right)+{\sum }_{i=0}^{\infty }{\Omega }_{i}\left(\tau \right){u}_{t-i}$$

Following Koop et al. (1996) and Pesaran and Shin (1998), the generalized forecast error variance decomposition (GFEVD) with a forecast horizon $H$ is specified as:

9 $${\Theta }_{ij}^{g}\left(H\right)=\frac{\sum {\left(\tau \right)}_{jj}^{-1}{\sum }_{h=0}^{H-1}{\left({e}_{i}^{^{\prime}}{\Omega }_{h}\left(\tau \right)\sum \left(\tau \right){e}_{j}\right)}^{2}}{{\sum }_{h=0}^{H-1}\left({e}_{i}^{^{\prime}}{\Omega }_{h}\left(\tau \right)\sum \left(\tau \right){\Omega }_{h}{\left(\tau \right)}^{^{\prime}}{e}_{i}\right)},$$

where ${e}_{i}$ represents a zero vector with unity at the $i$th position. The normalization of each element in the decomposition matrix is

10 $${\tilde{\Theta }}_{ij}^{g} \left( H \right) = \frac{{{\Theta }_{ij}^{g} \left( H \right)}}{{\mathop \sum \nolimits_{j = 1}^{k} {\Theta }_{ij}^{g} \left( H \right)}}{ }\,\,\,\,{\text{Where}} \,\,\,\, \mathop \sum \limits_{j = 1}^{k} {\tilde{\Theta }}_{ij}^{g} = 1\quad {\text{and}}\quad \mathop \sum \limits_{i,j = 1}^{k} {\tilde{\Theta }}_{ij}^{g} \left( H \right) = 1$$

Following Diebold and Yilmaz (2012, 2014), the various measures of connectedness at the $\tau$th conditional quantile can be estimated using the GFEVD method. Specifically, the total connectedness index $\left(TCI\right)$ measures the total connectedness effect in a system at the $\tau$th quantile, specified as follows:

11 $$TCI\left(\tau \right)=\frac{{\sum }_{i-1}^{k}{\sum }_{j=1,i\ne j}^{k}{\widetilde{\Theta }}_{ij}^{g}\left(\tau \right)}{{\sum }_{i-1}^{k}{\sum }_{j=1}^{k}{\widetilde{\Theta }}_{ij}^{g}\left(\tau \right)}\times 100$$

The *“TO”* directional connectedness index from index $i$ to all indices $j$ at quantile $\left(\tau \right)$ is:

12 $${TO}_{i\to j}\left(\tau \right)=\frac{{\sum }_{j=1, i\ne j}^{k}{\widetilde{\Theta }}_{ji}^{g}\left(\tau \right)}{{\sum }_{j=1}^{k}{\widetilde{\Theta }}_{ji}^{g}\left(\tau \right)}\times 100$$

The “*FROM*” directional connectedness index from all indices $j$ to index $i$ at quantile $\left(\tau \right)$ is:

13 $${FROM}_{i\leftarrow j}\left(\tau \right)=\frac{{\sum }_{j=1, i\ne j}^{k}{\widetilde{\Theta }}_{ij}^{g}\left(\tau \right)}{{\sum }_{j=1}^{k}{\widetilde{\Theta }}_{ij}^{g}\left(\tau \right)}\times 100$$

The “*NET*” directional connectedness index at quantile $\left(\tau \right)$ is:

14 $${NET}_{i}\left(\tau \right)={TO}_{i\to j}\left(\tau \right)-{FROM}_{j\leftarrow i}\left(\tau \right)$$

A positive (negative) value of ${NET}_{i}\left(\tau \right)$ identifies a net-transmitter (net-recipient) from the other markets. In practice, the dynamics of TCI are estimated on a QVAR with a 200-day window, a lag order of 1, (selected using the Bayesian Information Criterion), and a forecast horizon of 10.

## Empirical results

### Cross-quantilogram directional spillovers

**Fig. 4:**
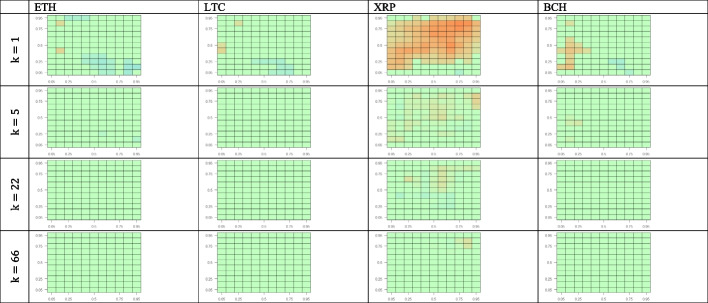
Cross-quantilogram heatmaps from BTC to other cryptocurrencies. *Notes*: This figure captures the cross-quantilogram dependence between cryptocurrencies for lags 1, 5, 22, and 66. Each cell in the heatmap corresponds to a cross-quantilogram for a specific pair of quantiles. The color scale at the bottom indicates the values of the cross-quantilograms. Any insignificant cross-quantilogram is set to 0, indicated with green in the heat maps

We estimate the CQ for 11 quantiles of the returns for BTC returns compared with those of the four other cryptocurrencies. Figure 4 shows the results for all possible quantile combinations. We test the predictability of crypto-market returns using the BTC returns for a set of lags (i.e., 1, 5, 22, and 66 working days). This approach is in line with Han et al. (2016), who consider up to 60 lags, and Jiang et al. (2016), who investigate up to 20 lags while estimating the CQ model with daily frequency time series.

We begin our visual inspection of Fig. 4 by analyzing the one-day lag. We observe that among the four cryptocurrencies, BTC returns positively predict only XRP returns. This predictability is most prominent in the medium–high quantile quadrant of the BTC–XPR panel. Additionally, the green shown in the lowest quantile of the BTC returns indicates that under bearish market conditions, XPR returns are invariant or very weakly sensitive to BTC returns. For the ETH and LTC panels, the blue color in the limited areas reveals that BTC returns in the medium–high quantiles are a weak negative predictor for ETH and LTC low quantile returns. Regarding BCH, we observe that under bearish market conditions BTC returns are a weak positive predictor of BCH performance.

Regarding the outcomes for 5-, 22-, and 66-day lags, we observe that the directional predictability largely disappears, becoming weaker as the lag increases for all cryptocurrencies considered. In summary, the cross-quantilogram heatmaps *from* BTC *to* other cryptocurrency markets do not reveal relevant asymmetric dependencies between BTC and the other cryptocurrencies’ returns except for the BTC–XRP one-day lag case, where BTC returns serve as a positive predictor for XPR’s medium–high quantile performance in all three states of the BTC market.

**Fig. 5:**
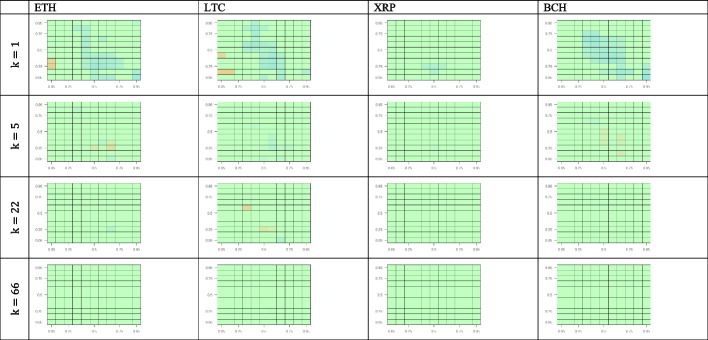
Cross-quantilogram heatmaps from other cryptocurrencies to BTC. *Notes*: See Fig. 4

Figure 5 presents cross-quantilogram heatmaps *from* other cryptocurrency markets *to* BTC. For the one-day lag, there is scattered evidence of weak (blue) negative predictability from three cryptocurrencies (ETH, LTC, and BCH) to BTC, mostly concentrated within the mid-quantiles corresponding to normal crypto-market conditions. In bearish and bullish market conditions, even this weak predictability vanishes. The same happens with larger lags, as the predominantly green cross-quantilogram panels for all the cryptocurrencies indicate an absence of asymmetric directional dependences between ETH, LTC, XRP, and BCH, as well as BTC.

**Fig. 6:**
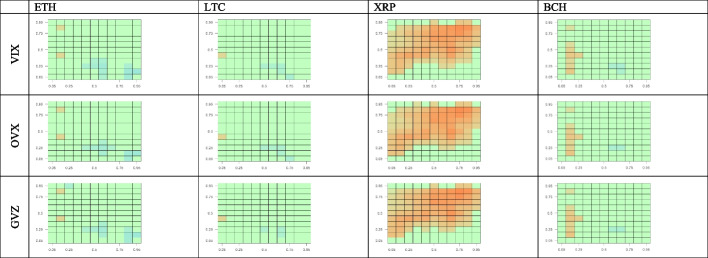
Cross-quantilogram heatmaps from BTC to other cryptocurrencies after controlling for volatility indices. *Notes*: See Fig. 4

Figure 6 shows the effects of BTC on the four other cryptocurrencies after controlling for uncertainties proxied by the equity, oil, and gold volatility indices (VIX, OVX, and GVZ, respectively). Figure 6 provides a comprehensive view of the directional predictability for all possible combinations of cross-quantiles of the returns for BTC and the four other cryptocurrencies. Here, we analyze only the one-day lag. Similar to the uncontrolled case, BTC returns positively predict XRP returns controlling for all volatilities. The strongest degree of predictability is consistently observed in the mid-high quantile quadrant, representing normal and bullish BTC market conditions. For the other cryptocurrencies, we do not see directional dependencies from BTC, except for BCH, where BTC serves as a rather weak positive predictor, although only for low quantiles corresponding to bearish BTC market conditions. Therefore, our results are robust and consistent with those obtained without controlling for stock, oil, and gold volatilities, as presented in Fig. 4.

**Fig. 7:**
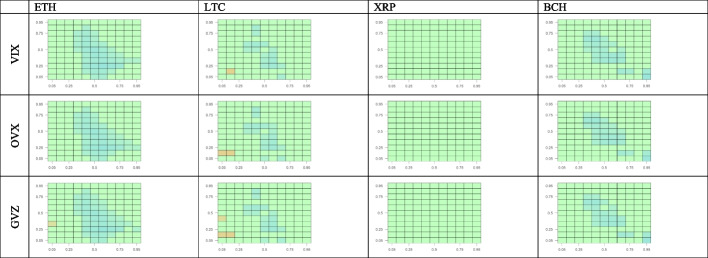
Cross-quantilogram heatmaps from other cryptocurrency markets to BTC after controlling for volatility indices. *Notes*: See Fig. 4

Figure 7 shows the effects of the four cryptocurrencies on BTC after controlling for uncertainties proxied by the equity, oil, and gold volatility indices. The one-day-lag cross-quantilogram heatmaps from the four other cryptocurrencies to BTC reveal a slight negative predictability under normal conditions in the ETH, LTC, and BCH markets. This is consistent with the respective uncontrolled cross-quantilograms presented in Fig. 5. Additionally, as shown in Fig. 7, no significant effect of XRP on BTC is observed, as indicated by the green color of the three XRP–BTC panels. This finding is somewhat consistent with the conclusion from Fig. 4 that BTC is a positive predictor of XRP for mid-high XRP quantiles but has no significant effects on low XRP quantiles.

In summary, we find some significant asymmetric dependence between the BTC and XRP markets for a one-day lag and show that predictability vanishes with higher lags. However, the effects between BTC and ETH, LTC, and BCH, are insignificant in both directions, independent of whether or not we control for equity, oil, and gold volatilities.

### Total quantile connectedness

Table 2 presents the overall connectedness results across the three quantiles (i.e., 0.05, 0.5, and 0.95), which correspond to bearish, normal, and bullish market conditions, respectively. Bearish and bullish conditions are referred to as extreme market states. Specifically, the values on the principal diagonal represent the own-shock contribution of an asset to its forecast error variance. The off-diagonal elements are shock spillovers from sources other than their own shocks, with the values along the rows representing the shocks transmitted by one variable to another and those in the columns representing the shocks received by one variable from others.

**Table 2:** Estimates of quantile spillovers between cryptocurrency returns and volatility indices

	BTC	ETH	LTC	XRP	BCH	VIX	OVX	GVZ	FROM
*Panel A: Lower quantile *$\left(\tau =0.05\right)$									
BTC	17.24	15.29	15.12	12.52	14.61	7.82	8.02	9.37	82.76
ETH	15.02	17.33	15.46	12.75	14.69	7.86	7.75	9.14	82.67
LTC	14.99	15.39	17.37	12.76	15.22	7.63	7.55	9.09	82.63
XRP	13.48	13.68	14.12	18.43	13.41	8.66	8.63	9.59	81.57
BCH	14.73	15.31	15.54	12.87	17.1	7.84	7.58	9.03	82.9
VIX	9.93	9.76	9.7	9.7	9.28	22.84	13.76	15.04	77.16
OVX	10.26	10.11	10.08	10.16	9.63	14.03	22.31	13.41	77.69
GVZ	10.77	10.54	10.71	10.5	10.22	13.89	12.2	21.17	78.83
TO	89.18	90.08	90.73	81.25	87.06	67.74	65.49	74.66	646.2
ALL	106.42	107.41	108.1	99.68	104.16	90.58	87.81	95.84	TCI
NET	6.42	7.41	8.1	− 0.32	4.16	− 9.42	− 12.19	− 4.16	80.78
*Panel B: Median quantile *$\left(\tau =0.5\right)$									
BTC	30.69	20.4	19.59	8.1	18.26	2.01	0.71	0.24	69.31
ETH	19.43	29.44	20.34	9.16	19	1.88	0.56	0.18	70.56
LTC	18.66	20.34	29.09	8.96	20.48	1.83	0.5	0.14	70.91
XRP	11.8	14.11	13.88	43.66	13.61	1.68	0.68	0.58	56.34
BCH	17.7	19.44	20.95	9.17	30.4	1.67	0.47	0.19	69.6
VIX	3.66	3.5	3.48	2.19	3.19	63.44	8.81	11.72	36.56
OVX	1.54	1.29	1.17	0.85	1.07	10.7	76.54	6.84	23.46
GVZ	0.51	0.34	0.28	0.94	0.45	13.8	6.77	76.91	23.09
TO	73.3	79.42	79.7	39.38	76.08	33.56	18.5	19.89	419.83
ALL	103.99	108.87	108.78	83.03	106.48	97	95.04	96.81	TCI
NET	3.99	8.87	8.78	− 16.97	6.48	− 3	− 4.96	− 3.19	52.48
*Panel C: Upper quantile *$\left(\tau =0.95\right)$									
BTC	16.79	14.6	14.45	11.68	14.99	8.29	9.26	9.93	83.21
ETH	14.48	16.95	14.74	11.97	15.01	8.19	8.81	9.83	83.05
LTC	14.39	14.55	16.45	11.59	15.24	8.27	9.47	10.05	83.55
XRP	13.35	13.7	13.65	18.06	13.99	8.5	8.92	9.83	81.94
BCH	14.55	14.47	14.68	11.87	17.71	8.03	9.01	9.68	82.29
VIX	10.52	10.45	10.59	9.7	10.8	19.17	13.96	14.82	80.83
OVX	10.62	10.51	10.74	9.36	10.77	13.83	20.39	13.77	79.61
GVZ	11.11	11.07	10.8	9.49	11.15	13.75	12.99	19.65	80.35
TO	89.02	89.35	89.65	75.65	91.96	68.87	72.42	77.92	654.83
ALL	105.82	106.3	106.09	93.71	109.67	88.04	92.82	97.56	TCI
NET	5.82	6.3	6.09	− 6.29	9.67	− 11.96	− 7.18	− 2.44	81.85

The connectedness table is based on a quantile VAR model with lag length of order 1 (BIC) and a 10-step-ahead forecast

Panel A of Table 2 shows that for the lower quantile (0.05), all markets are significant transmitters and receivers of spillover. All of the cryptocurrencies in the study play a leading role in the network, transmitting the highest spillover within a range of 81.25% (XRP) to 90.73% (LTC). The three volatility indices transmit weaker spillover, ranging from 74.66% (GVZ) to 65.49% (OVX). The differences in these contributions to the system are further revealed through the net spillover strength. In terms of net contributions to the network, four cryptocurrencies (all but XRP) are net transmitters of system innovations, whereas all three volatility indices are net recipients. The two highest positive net spillover rates of 8.1% and 7.41% are provided by LTC and ETH, respectively, while the two largest negative spillover rates of − 12.19% and − 9.42% are observed for the OVX and VIX indices, respectively.

The differences in spillover transmissions are notable within and between the two subgroups of variables in the system, i.e., cryptocurrencies and volatility indices. By analyzing the nondiagonal elements of the 5 × 5 top left quadrant and the 3 × 3 lower right quadrant, and comparing them with the 5 × 3 top right quadrant and the 3 × 5 lower left quadrant, we observe that the shock spillover between subgroups (8.37% from volatility indices to cryptocurrencies and 10.09% from crypto to volatility indices, on average) is considerably weaker than the spillover within the subgroups (14.34% within cryptocurrencies and 13.72% within volatility indices, on average), even when diagonal elements are not considered.

The TCI is quite high (80.78%) under bearish market conditions, indicating that the overall system in general, and cryptocurrencies in particular, are highly vulnerable to risks during periods of unexpected negative news in a bearish market. Calm/normal market conditions (the 0.5 quantile) do not change the pattern of risk transmission across these two subgroups (see Panel B in Table 2). However, there are differences in the depth of the transmission or receipt of shocks, mostly resulting in weaker pairwise spillover effects across the system. Notably, the shock spillover between subgroups becomes practically insignificant under normal market conditions (averaging 0.88%, from volatility indices to cryptocurrencies and 1.63% from crypto to volatility indices). This indicates that the transmission of innovation between the cryptocurrencies and volatility indices does not occur under normal market conditions, implying that these two subsystems may be viewed independently. The values of all diagonal elements of the connectedness matrix increase considerably, especially for the volatility indices (from 63.44% for VIX to 76.91% for GVZ). The principal diagonal elements represent own-shock contributions, thereby showing that idiosyncratic features in system behavior are much stronger under normal market conditions compared to bearish and bullish markets, in which the overall market’s tendency dominates the individual drivers of cryptocurrencies and equity, oil, and gold volatilities.

The TCI under normal market conditions is rather modest at 52.48%, and well below the values observed for bearish and bullish markets, at 80.78% and 81.85%, respectively. This indicates that in general, the entire system is not highly susceptible to idiosyncratic risks, nor is it expected to amplify those risks into systemic risk events. We note that in a bullish market, represented by the upper quantile (0.95), the pattern of risk transmission among the markets is similar to those seen in bearish and normal markets. However, as shown in Panel C of Table 2, there are differences in the higher strengths of pairwise spillover effects across the system, similar to those seen under bearish market conditions (Panel A, Table 2), but different from normal market conditions (Panel B, Table 2). For bearish markets, we observe that the shock spillover between subgroups (averaging 8.03% from volatility indices to cryptocurrencies, and 10.51% from crypto-markets to volatility indices) is considerably weaker than the spillover within subgroups (13.89% within cryptocurrencies and 13.85% within volatility indices, on average), even without considering the principal diagonal elements that represent the own-shock contribution of an asset to its forecast error variance. One exception is that under normal market conditions, BCH becomes a major net transmitter of shocks (9.67%) as it transmits more information (91.96%) than it receives (82.29%). Similar to bearish market conditions where the TCI is 80.78%, the TCI under bullish market conditions is even higher at 81.85%, meaning that the overall system, particularly the cryptocurrencies, are highly susceptible to unexpected news and market risks, and market stability is compromised and weakened under extreme market conditions corresponding to both bear and bull markets.

These results provide interesting insights. The risk transmission among markets tends to be significantly affected by market conditions. In particular, global crises that result in bear markets create systemic connectedness is-à-vis the normal state of the market. However, in all market conditions cryptocurrencies show a leadership influence over the volatility indices. This is to be expected, as the volatility indices for equities, crude oil, and gold reflect realized, up-to-the-date volatility levels, whereas cryptocurrency prices are intrinsically forward-looking as they incorporate current market expectations regarding the future performance of respective crypto-assets. These findings are consistent with the vulnerability of cryptocurrencies to spillover in the system, especially during global crises, as reported in previous studies (Umar and Gubareva 2020; Hsu et al. 2021; Zhang and He 2021; Attarzadeh and Balcilar 2022; Naeem et al. 2022).

Intensifying risks under extreme market conditions suggests that forming an investment portfolio containing cryptocurrencies is riskier during periods of market volatility, regardless of whether the volatility is due to positive or negative news, compared with periods when the market is free from unexpected exogenous news. Total connectedness peaks in extreme market conditions, whereas we see a lower local minimum under normal market conditions. Additionally, we show it is essential for cryptocurrency market investors to closely monitor systemic risks within the crypto ecosystem, which remain mostly uncaptured by the equity, crude oil, and gold volatility indices. Nonetheless, volatility-based financial instruments can potentially provide suitable hedges for crypto-investors, as cryptocurrency and volatility markets are shown to be insignificantly (weakly) connected under normal (extreme) market conditions. Further research on forward-looking cryptocurrency portfolio management and downside risk hedging is desirable.

### Time-varying connectedness analysis

**Fig. 8:**
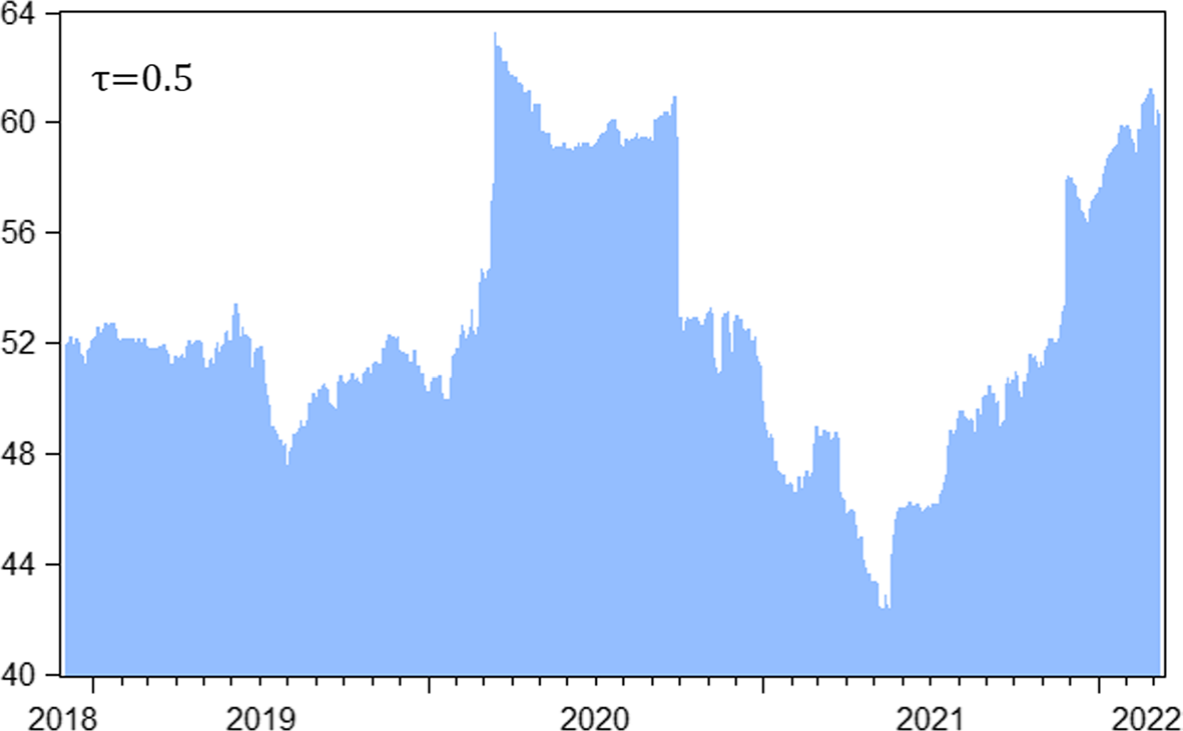
Total spillover in the medium quantile VAR (median quantile $\tau =0.5$). *Notes*: Total connectedness index (TCI) computed using a rolling window of 200 days and 10 step-ahead forecast horizons

A time-varying connectedness analysis not only reveals the time dynamics of spillovers but also detects performance diversity during both calm and tumultuous periods in financial markets. Thus, we take the additional step of estimating the total dynamic connectedness across the median and extreme quantiles. Figure 8 shows the time-varying total spillover for the median (0.5) quantile. We observe moderate connectedness in 2018–2019, near 50%. It abruptly increases in the first quarter of 2020, rising above 60% during the rapid global spread of COVID-19 that caused major sell-offs in financial markets. This finding is consistent with previous studies that report stronger connectedness across financial markets during crises (Adekoya and Oliyide 2021; Umar et al. 2021a; 2022b; Yousaf et al. 2022). At the end of the third quarter of 2020, connectedness showed a similarly abrupt decline, returning to pre-COVID-19 levels. Through a visual inspection of Fig. 8 we can compare the impact of the pandemic on total connectedness to the “rectangular” unit impulse signal function () representing the COVID-19-triggered impact, which is added to the otherwise “unperturbed” dynamics in our cryptocurrencies-plus-volatilities system. We ascribe the abrupt return to the pre-COVID patterns of connectedness that occurred in the second half of 2020 to good news regarding the development of COVID-19 vaccines, allowing the pandemic to be contained (Rouatbi et al. 2021). After returning to pre-COVID-19 levels, connectedness continued to decline, reaching an all-time low of approximately 40% by the end of the first half of 2021. Since then, it has commenced its uptrend to reach 60%, caused by elevated global uncertainty related to the military conflict in Ukraine and rising inflation worldwide.

**Fig. 9:**
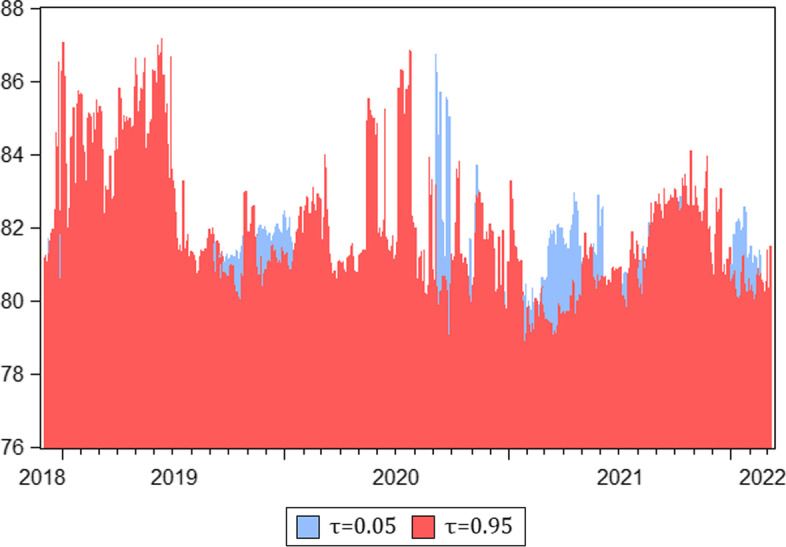
Dynamics of total connectedness in the extreme lower ($\tau =0.05$) and upper ($\tau =0.95$) quantile VAR

Figure 9 shows the dynamic total connectedness results for the extreme quantiles. Consistent with evidence from extant literature (Saeed et al. 2020; Mensi et al. 2021a; Khalfaoui et al. 2022) we observe higher spillover at the extremes compared with the median quantile. The lowest total spillover for the extreme quantiles was above 77% in mid-2020, while the highest total spillover in the median quantile is below 65%. The maximum total spillover exceeds 87% in some instances for both the lower and upper quantiles. Unlike the low volatility of connectedness in the median quantile during the pre-COVID-19 period, at roughly 50%, upper quantile connectedness increased substantially to 87% in 2019, under bullish market conditions, and then in the second quarter of 2020, when markets were starting to recover from the low point of the COVID-19 crisis. We find that total connectedness is higher for the upper quantile compared with the lower quantile across most periods, with a few exceptions observed predominantly from the second half of 2020 onwards.

**Fig. 10:**
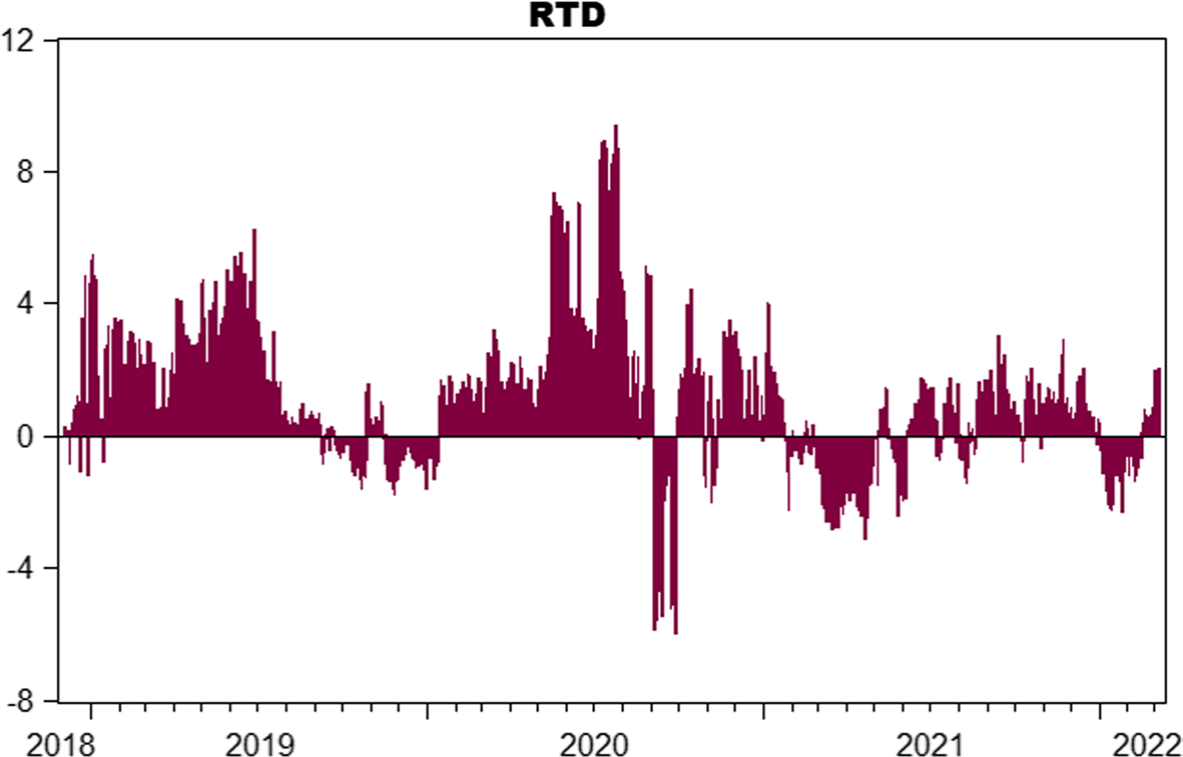
Relative tail dependence $\left({TCI}_{\tau =0.95}-{TCI}_{\tau =0.05}\right).$ *Notes*: This figure shows relative tail dependence calculated as the difference between the TCI at the 95th quantile and the 5th quantile. The negative (positive) value indicates a strong dependence on the lower (upper) quantile

For a clearer comparison, Fig. 10 plots the differences between the total connectedness of the upper and lower quantiles. A positive value suggests stronger connectedness in the upper quantile, whereas a negative value indicates the opposite. Consistent with our observations above, positive values prevail in most periods, indicating that connectedness in the upper quantile is generally dominant, while connectedness in the lower quantile dominates only during certain periods. We conclude that crypto-investors are more sensitive to unexpected positive shocks, regardless of whether the market is bearish, normal, or bullish. This is generally consistent with the mostly overly-optimistic sentiment observed in cryptocurrency markets over the four years covered in this study, which could be compared to the so-called irrational exuberance of markets before the global financial crisis of 2007–2008, which results in so-called “panic-buying” due to the fear of missing out on profitable investment opportunities and underestimates the risks involved. Notably, the crypto-crash in May–June 2022 may have altered crypto-investors’ euphoric attitudes. Thus, further research in this field is necessary to assess whether crypto-investors continue to be more sensitive to unexpected good shocks or have become more susceptible to unexpected bad news.

Overall, our findings suggest the importance of considering extreme market situations and timing when analyzing connectedness and, by implication, spillover of shocks among cryptocurrencies and traditional financial markets. Contagion effects under extreme market conditions are stronger than average risk transmissions in normal markets, which is of great importance to investors. Hence, when structuring an investment portfolio based on exposure to cryptocurrency, hedge strategies should be designed using financial products that have a weak or no connection to crypto-assets, such as the volatility-based instruments in our study. Moreover, intensifying risks during periods of either good or bad unexpected market news, compared to under normal market conventions call for another level of care in making investment decisions.

**Fig. 11:**
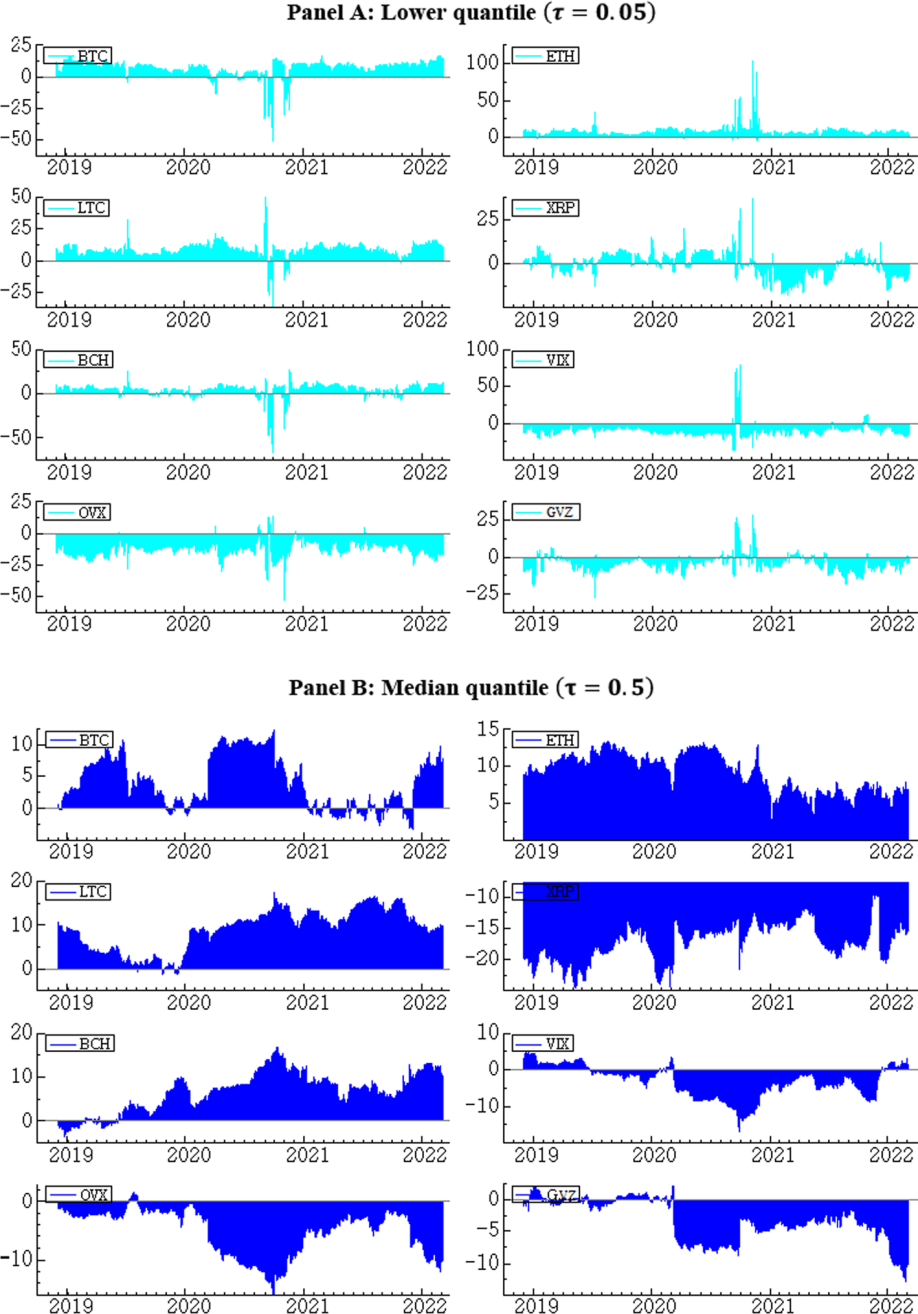

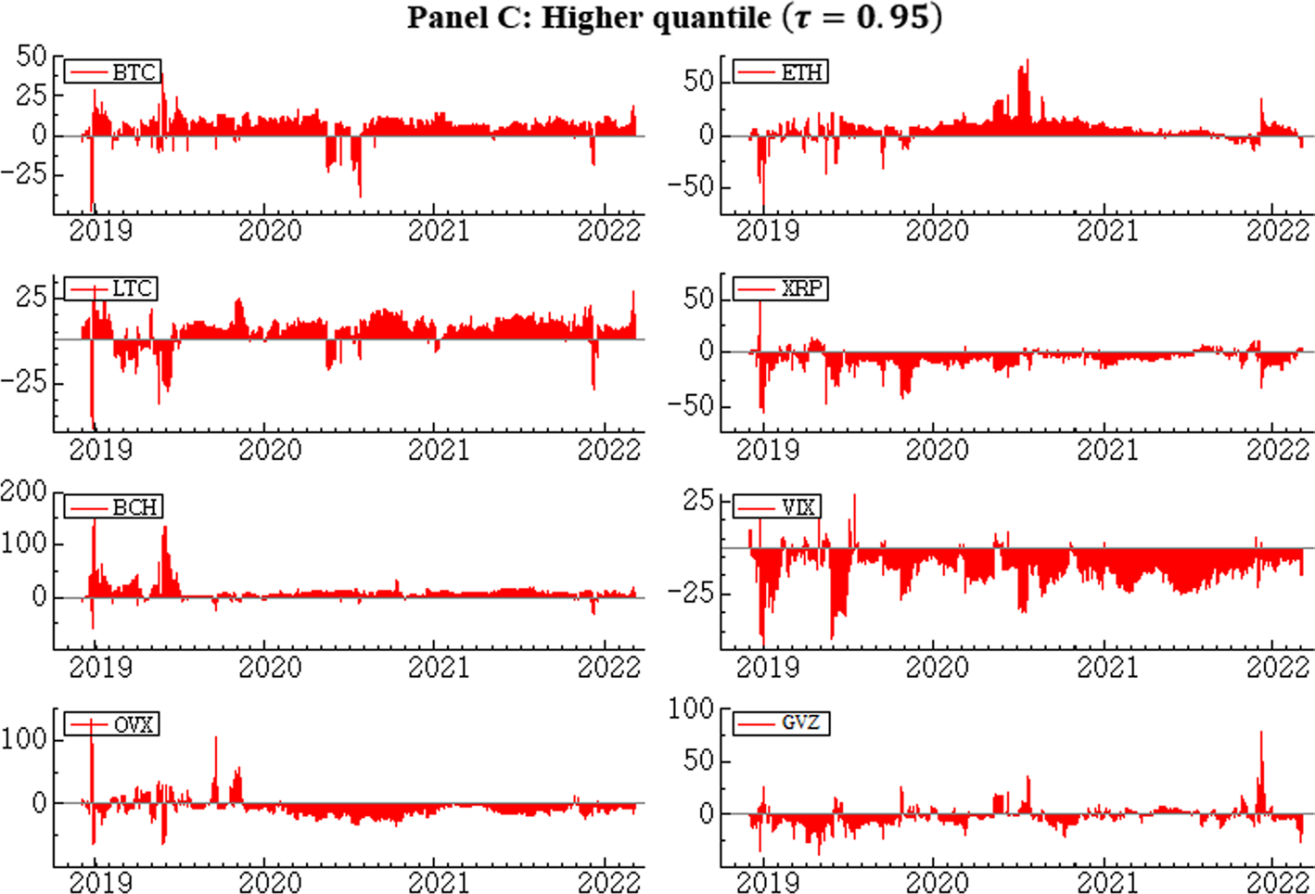
Dynamic net spillover in quantile VAR of cryptocurrency and volatility indices

Next, we examine the time-varying net spillover of each series in the median and extreme quantiles. First, we show the time-varying net spillover effects in the lower quantile (Fig. 11, Panel A). Consistent with Panel A of Table 2, the net spillover is mostly positive for BTC, ETH, LTC, and BCH, indicating that these markets are predominantly net transmitters, while net spillover is mostly negative for XRP and the three volatility indices, indicating that these markets are net recipients. Nevertheless, major crises, specifically the COVID-19-related market crisis in the first half of 2020, are associated with high net spillovers in all markets. Moreover, with the exception of XRP the net connectedness values skew more strongly toward the positive axis for cryptocurrencies, implying that at the left tail these assets tend to transmit more shocks than they receive. The opposite holds for all three volatility indices considered.

Continuing to the median quantile (Fig. 11, Panel B), we see that the roles of transmitters and receivers are unchanged, but in line with Panel B of Table 2 and Figs. 8 and 9, the strength of spillover for the median quantile is, on average, weaker than in the lower and upper quantiles.

Finally, Panel C of Fig. 11 shows the time-varying net spillover in the upper quantile. The net transmitting and receiving variables in the system remain unchanged; however, the spillover strength is substantially higher than in the median quantile, and similar to what is seen in the lower quantile. These findings are consistent with Panel C of Table 2 and the conclusion of the comparative analysis across all panels of Table 2. Our findings imply that at the extreme quantiles, especially compared to the median quantile, risk transmission and contagion effects are at their maximum, on average. Therefore, high net spillover in the system is indicative of both boom and bust periods associated with major crises and recoveries from those crises, and signifies that systemic risk contagion is amplified under bullish and bearish market conditions.

### Connectedness network

**Fig. 12:**
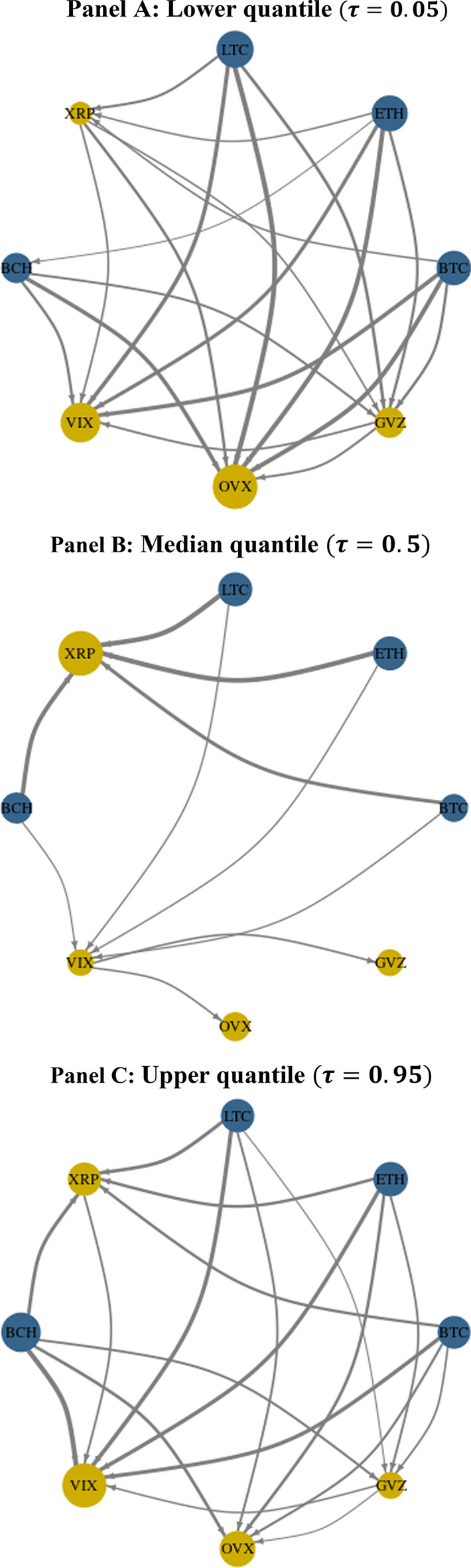
Net pairwise directional connectedness network at different quantiles. *Notes*: Blue (yellow) nodes indicate net transmitters (recipients) of shocks. Vertices are weighted using the averaged net pairwise directional connectedness measures. The size of the nodes represents the weighted average net total directional connectedness

We further explore the connectedness network by visually inspecting the net pairwise directional spillover between the five cryptocurrency markets and three volatility indices.^2^ The node size indicates the magnitude of the net transmission or receipt of shocks. The arrows indicate the direction of the innovation flows from the blue nodes, which represent net contributing variables, to the yellow nodes, which denote variables that are net shock receivers. The width of the arrows represents the strength of the transmitter–receiver interaction between the two nodes. Figure 12 plots 27 pairwise edges with eight nodes, then removes edge weights that are less than 1 among the 27 pairwise connections.

Panel A of Fig. 12 shows that in the lower quantile, with the exception of XRP the cryptocurrencies are net pairwise transmitters of system shocks. However, these four net-transmitting cryptocurrencies notably do not transmit or receive shocks among themselves, other than the innovation transmission from ETH to BCH. In addition, we find that XRP, despite being a net receiver, transmits shocks to all three volatility indices. Regarding the interaction among the volatility indices, the gold volatility index transmits shocks to the other two. The OVX node is the largest, indicating that the crude oil volatility index is the most important net receiver of system shocks in the lower quantile, which captures bearish market conditions.

In Panel B, which shows the results for the median quantile, we observe that the receiver and transmitter roles remain unchanged compared with Panel A; however, the relative strengths of the nodes are different. For example, the largest receiver is now XRP, not OVX. One notable observation is that the topology of system connectedness in the median quantile is much simpler than in the lower quantile (Panel A). Here, the four net-transmitting cryptocurrencies do not transmit or receive any shocks from each other, and transmit innovations to only two nodes: XRP and VIX. In turn, VIX transmits shocks to the other two volatility indices and is now the only contributor to innovations for both OVX and GVZ. In general, in the median quantile, corresponding to normal market conditions, the system is less connected and therefore less vulnerable to systemic risk transmission and less likely to cause market contagion.

Panel C shows that in the upper quantile, system connectedness is substantially more complex than in the median quantile (Panel B), but slightly simpler than in the lower quantile (see Panel A). The receiver and transmitter roles remain unchanged compared with the other quantiles; however, certain links between the nodes are now absent. For example, in contrast to the lower quantile case, XRP directly transmits shocks only to VIX, which is now the largest net receiver but does not directly influence either OVX or GVZ.

The pattern of pairwise connectedness differs across the quantiles, but regardless of market conditions the four cryptocurrencies, BTC, LTC, ETH, and BCH, are net pairwise transmitters of shocks to XRP and the volatility indices. This reveals the prominent role of the returns for these dominant cryptocurrencies in terms of market volatility dynamics. We also find that the system is more connected under extreme market conditions and, hence, more vulnerable to contagion and systemic risk transmission. These results offer insights for a range of market participants and could be used by crypto-investors and portfolio managers in assessing hedge effectiveness of joint exposures to cryptocurrency and volatility markets.

## Conclusions

This study employs the cross-quantilogram method and a quantile connectedness approach to examine tail spillover between five cryptocurrencies (BTC, ETH, LTC, XRP, and BCH), and three CBOE uncertainty indices (VIX, OVX, and GVZ), with a special focus on the effects of the COVID-19 pandemic on time-varying network connectedness and relative tail dependence. We also analyze the connectedness network interactions based on the magnitude and direction of pairwise correlations, including how the pandemic affected these interactions.

To assess directional spillovers, we estimate and visually inspect cross-quantilograms of the returns of BTC compared with returns of the four other cryptocurrencies. We find some significant asymmetric dependence between the BTC and XRP markets under a one-day lag and show that predictability vanishes with an increase in lag parameters. However, the effects in either direction between BTC, as well as ETH, LTC, and BCH, are rather insignificant, whether or not we control for equity, oil, and gold volatilities.

We estimate overall connectedness results across three quantiles (i.e., 0.05, 0.5, and 0.95), corresponding to bearish, normal, and bullish market conditions, respectively. The TCI under normal market conditions is moderate (52.48%), and well below the values observed for the quantiles corresponding to bearish and bullish markets (80.78% and 81.85%, respectively). Thus, we provide evidence that risk transmission among cryptocurrency and volatility markets tends to be significantly affected by market conditions. In particular, global crises that result in bear markets, making the systemic connectedness to augment vis-à-vis the normal state of the market. We also show that the cryptocurrencies have a leadership influence over the volatility indices under all market conditions. Our results have important implications for volatility-based financial instruments, which can potentially provide suitable hedges for cryptocurrency investors. For example, the results suggest a potential hedge for a long position in a cryptocurrency using a long position in a VIX futures contract. This setup is common for hedging equity investments against downturns, and there are several instances that show losses in equity portfolios during turbulent times are fully offset by gains in the VIX futures contracts (Ryvkin 2019). Given that in times of crisis, risk aversion affects both traditional and crypto markets, which share common shocks, the VIX futures contracts could be used to hedge cryptocurrency investments. Moreover, other volatility benchmarks, such as GVX (gold) or CVX (cryptocurrency) indices computed from option prices, may offer valuable hedging strategies to cryptocurrency investors (Woebbeking 2021). In particular, we show that cryptocurrency and volatility markets are insignificantly (weakly) connected under normal (extreme) market conditions. Further research on forward-looking cryptocurrency portfolio management and downside risk hedging is needed to corroborate what our study suggests.

To investigate time-varying differences in spillover effects during calm and volatile markets, we estimate the dynamic total connectedness across median and extreme quantiles. We find that total connectedness was moderate, near 50%, in 2018–2019, and then abruptly climbed to above 60% in the first quarter of 2020, during the rapid global spread of COVID-19. This finding is consistent with previous studies that show financial markets often display stronger connectedness during crises. Analyzing dynamic total connectedness for the extreme quantiles, we find that spillover is higher at the extremes than at the median quantile, which is consistent with evidence from previous research. In addition, we find that total connectedness is higher for the upper quantile than for the lower quantile across most of our sample.

Our connectedness network analysis reveals that the pattern of pairwise connectedness varies across quantiles. However, regardless of market conditions, four cryptocurrencies (BTC, LTC, ETH, and BCH) are net pairwise transmitters of shocks to XRP and to the volatility indices. This reveals the leading role of the returns of the most prominent cryptocurrencies in explaining market volatility dynamics. We also show that the system is more connected under extreme market conditions and is therefore more vulnerable to contagion and systemic risk transmission. Our results offer insights for a range of market participants and should be carefully considered by crypto-investors and portfolio managers when assessing the hedging effectiveness of joint exposures to cryptocurrency and volatility markets.

Our findings provide relevant insights for both policymakers and crypto-market participants as we show that spillover effects are time-varying and asymmetric. Specifically, during major crises investors may use knowledge of crypto-market patterns and the volatility of major traditional markets to hedge their exposure to cryptocurrencies. The findings provided through our research on directional and pairwise connectedness and network spillover topology are also useful for efficient portfolio management.

Our findings on dynamic volatility connectedness across diverse quantiles also have implications for financial stability and monetary policy by providing important information about the risk profiles of the cryptocurrencies we analyzed, in particular their inherently high volatility, due to the absence of any underlying fundamental value among others factors. It is important for market regulators to continuously monitor ongoing developments in the cryptocurrency markets and their links to traditional financial markets and the real economy. The volatility connectedness revealed in this study suggests that policymakers should conduct thorough surveillance to detect and address system-wide vulnerabilities. This is especially important given the risk that cryptocurrencies may be used for capital flight. As they are highly speculative assets, cryptocurrencies may also amplify flight-to-quality and flight-to-safety events, endangering financial stability (Gubareva et al. 2022). Overall, our findings regarding tail spillover effects between cryptocurrencies and volatility in the gold, oil, and equity markets indicates the need for policymakers to develop informed policies governing this arena.

In light of recent turmoil in crypto-asset markets, regulators are become increasingly concerned with regulating this market, including platforms that facilitate trading in crypto-instruments. For example, in July 2022 the Financial Stability Board (FSB) published a statement on international regulation and supervision of crypto-asset activities, outlining the work undertaken by the FSB and international standard-setting bodies to address the potential financial stability risks posed by crypto-assets, including so-called stablecoins (FSB 2022). In particular, the statement says that crypto-assets and markets must be subject to effective regulation and oversight that is commensurate with the risks they pose, at both domestic and international levels. This is especially important given the permissionless and pseudonymous design of rapidly growing DeFi platforms that create many challenges in terms of enforcing tax compliance, upholding anti-money laundering laws, and preventing financial malfeasance (Makarov and Schoar 2022). Moreover, the phenomenon of wash trading that is proliferating among crypto-asset markets amplifies the risks and volatility of digital instruments (Cong et al. 2022). Wash-trading fabricates volumes, distorts prices, amplifies return volatility and disguises true market conditions, creating an urgent need for regulators to develop adequate supervision for cryptocurrency and DeFi trading activities. The results of our research are timely and relevant given the many challenges faced by regulators of crypto-asset markets.

In summary, we note that while aggressive investors have generated large exposures to cryptocurrencies, studies analyzing crypto-asset portfolio dynamics across various quantiles are rare. Our research contributes to the body of literature by analyzing tail spillover using the cross-quantilogram method and quantile connectedness approach, providing relevant insights for managing portfolio risks in times of financial market crises such as the one that resulted from COVID-19 pandemic. Our research enhances the current understanding of the network connectedness and quantile interdependencies of cryptocurrencies and could help to improve financial stability.

This study could be extended by analyzing spillovers in high-order moments among diverse cryptocurrencies. It could also be extended by exploring cojumps in other crypto-assets, such as NFTs and DeFi, and by analyzing the high-frequency connectedness in jumps, kurtosis, and skewness between cryptocurrencies and volatility indices for, gold, oil, and equity markets. Future research could also examine the key factors driving the spillovers and connectedness among cryptocurrencies, volatility indices and potential safe-haven assets under different volatility regimes using both the MS-VAR model and the spillover index of Diebold and Yilmaz (2012).

## Data Availability

The datasets generated and/or analysed during the current study are not publicly available due to data security but are available from the corresponding author on reasonable request.

## Abbreviations

BTC: Bitcoin
ETH: Ethereum
LTC: Litecoin
XRP: Ripple
BCH: Bitcoin Cash
VIX: Stock volatility index (VIX)
OVX: Crude oil volatility index
GVZ: Gold volatility index
TCI: Total connectedness index
CQ: Cross-quantilogram
SB: Stationary bootstrap
PCQ: Partial cross-quantilogram
QVAR: Quantile vector autoregression
GFEVD: Generalized forecast error variance decomposition

## Footnotes

1 For more information on the market capitalization and trading volumes of cryptocurrencies, we refer the reader to the Coinmarket website, https://coinmarketcap.com/

2 We use the Connectedness Approach (https://davidgabauer.shinyapps.io/connectedness_approach/) to visualize net pairwise connectedness network graphs. Edges and node locations are determined using the R software’s “ConnectednessApproach”.

## References

[CR1] Adekoya OB, Oliyide JA (2021). How COVID-19 drives connectedness among commodity and financial markets: evidence from TVP-VAR and causality-in-quantiles techniques. Resour Policy.

[CR2] Agarwal V, Ruenzi S, Weigert F (2017). Tail risk in hedge funds: a unique view from portfolio holdings. J Financ Econ.

[CR3] Ando T, Greenwood-Nimmo M, Shin Y (2022). Quantile connectedness: modelling tail behaviour in the topology of financial networks. Manag Sci.

[CR4] Antonakakis N, Chatziantoniou I, Gabauer D (2019). Cryptocurrency market contagion: market uncertainty, market complexity, and dynamic portfolios. J Int Financ Mark Inst Money.

[CR5] Attarzadeh A, Balcilar M (2022). On the dynamic return and volatility connectedness of cryptocurrency, crude oil, clean energy, and stock markets: a time-varying analysis. Environ Sci Pollut Res.

[CR6] Bams D, Blanchard G, Honarvar I, Lehnert T (2017). Does oil and gold price uncertainty matter for the stock market?. J Empir Finance.

[CR7] Benlagha N, El Omari S (2022). Connectedness of stock markets with gold and oil: new evidence from COVID-19 pandemic. Finance Res Lett.

[CR8] Bianchi D, Babiak M, Dickerson A (2022). Trading volume and liquidity provision in cryptocurrency markets. J Bank Finance.

[CR9] Borri N (2019). Conditional tail-risk in cryptocurrency markets. J Empir Finance.

[CR10] Bossman A, Gubareva M (2023). Asymmetric impacts of geopolitical risk on stock markets: a comparative analysis of the E7 and G7 equities during the Russian-Ukrainian conflict. Heliyon.

[CR11] Bossman A, Gubareva M, Teplova T (2023). Asymmetric effects of geopolitical risk on major currencies: Russia-Ukraine tensions. Finance Res Lett.

[CR12] Bossman A, Gubareva M, Teplova T (2023). EU sectoral stocks amid geopolitical risk, market sentiment, and crude oil implied volatility: an asymmetric analysis of the Russia-Ukraine tensions. Resour Policy.

[CR13] Bouri E, Lau C, Lucey B, Roubaud D (2019). Trading volume and the predictability of return and volatility in the cryptocurrency market. Financ Res Lett.

[CR14] Bouri E, Saeed T, Vo XV, Roubaud D (2021). Quantile connectedness in the cryptocurrency market. J Int Financ Mark Inst Money.

[CR15] Dickey DA, Fuller WA (1979). Distribution of the estimators for autoregreesive time series with a unit root. J Am Stat Assoc.

[CR16] Diebold FX, Yilmaz K (2012). Better to give than to receive: predictive directional measurement of volatility spillovers. Int J Forecast.

[CR17] Diebold FX, Yilmaz K (2014). On the network topology of variance decompositions: measuring the connectedness of financial firms. J Econom.

[CR18] Fang F, Ventre C, Basios M, Kanthan L, Martinez-Rego D, Wu F, Li L (2022). Cryptocurrency trading: a comprehensive survey. Financ Innov.

[CR19] Fendel R, Neumann C (2021). Tail risk in the European sovereign bond market during the financial crises: detecting the influence of the European Central Bank. Glob Finance J.

[CR20] Ghorbel A, Loukil S, Bahloul W (2022). Connectedness between cryptocurrencies, gold and stock markets in the presence of the COVID-19 pandemic. Eur J Manag Bus Econ.

[CR21] Godil DI, Sarwat S, Sharif A, Jermsittiparsert K (2020). How oil prices, gold prices, uncertainty and risk impact Islamic and conventional stocks? Empirical evidence from QARDL technique. Resour Policy.

[CR22] Gubareva M, Umar Z, Teplova T, Vo XV (2022). Flights-to-quality from EM bonds to safe-haven US Treasury securities: a time-frequency analysis. Emerg Mark Financ Trade.

[CR23] Han H, Linton O, Oka T, Whang Y (2016). The cross-quantilogram: measuring quantile dependence and testing directional predictability between time series. J Econom.

[CR24] Hanif W, Mensi W, Gubareva M, Teplova T (2023). Impacts of COVID-19 on dynamic return and volatility spillovers between rare earth metals and renewable energy stock markets. Resour Policy.

[CR25] Happersberger D, Lohre H, Nolte I (2020). Estimating portfolio risk for tail risk protection strategies. Eur Financ Manag.

[CR26] Hoang T-H-V, Lean HH, Wong W-K (2015). Is gold good for portfolio diversification? A stochastic dominance analysis of the Paris stock exchange. Int Rev Financ Anal.

[CR27] Hsu S-H, Sheu C, Yoon J (2021). Risk spillovers between cryptocurrencies and traditional currencies and gold under different global economic conditions. N Am J Econ Finance.

[CR28] Jena S, Tiwari AK, Doğan B, Hammoudeh S (2020). Are the top six cryptocurrencies efficient? Evidence from time-varying long memory. Int J Finance Econ.

[CR29] Jiang H, Su JJ, Todorova N, Roca E (2016). Spillovers and directional predictability with a cross-quantilogram analysis: the case of US and Chinese agricultural futures. J Futures Mark.

[CR30] Kelly B, Jiang H (2014). Tail risk and asset prices. Rev Financ Stud.

[CR31] Khalfaoui R, Jabeur SB, Doğan B (2022). The spillover effects and connectedness among green commodities, bitcoins, and US stock markets: evidence from the quantile VAR network. J Environ Manag.

[CR32] Koop G, Pesaran MH, Potter SM (1996). Impulse response analysis in nonlinear multivariate models. J Econom.

[CR33] Kwiatkowski D, Phillips PCB, Schmidt P, Shin Y (1992). Testing the null hypothesis of stationarity against the alternative of unit root. J Econom.

[CR34] Lesame K, Bouri E, Gabauer D, Gupta R (2021). On the dynamics of international real-estate-investment trust-propagation mechanisms: evidence from time-varying return and volatility connectedness measures. Entropy (basel).

[CR35] Maghyereh A, Abdoh H (2022). COVID-19 and the volatility interlinkage between bitcoin and financial assets. Empir Econ.

[CR36] Maitra D, Rehman MU, Dash SR, Kang SH (2022). Do cryptocurrencies provide better hedging? Evidence from major equity markets during COVID-19 pandemic. N Am J Econ Finance.

[CR37] Mandaci PE, Cagli EC (2022). Herding intensity and volatility in cryptocurrency markets during the COVID-19. Finance Res Lett.

[CR38] Mensi W, Al Rababa’a AR, Vo XV, Kang SH (2021). Asymmetric spillover and network connectedness between crude oil, gold, and Chinese sector stock markets. Energy Econ.

[CR39] Mensi W, Al-Yahyaee KH, Idries Mohammad Wanas Al-Jarrah IMW, Vo XV, Kang SH (2021). Does volatility connectedness across major cryptocurrencies behave the same at different frequencies? A portfolio risk analysis. Int Rev Econ Finance.

[CR40] Mensi W, Vo XV, Kang SH (2022). COVID-19 pandemic’s impact on intraday volatility spillover between oil, gold, and stock markets. Econ Anal Policy.

[CR41] Naeem MA, Qureshi S, Arif M, Balli F (2021). Asymmetric relationship between gold and Islamic stocks in bearish, normal and bullish market conditions. Resour Policy.

[CR42] Naeem MA, Qureshi S, Rehman MU, Balli F (2022). COVID-19 and cryptocurrency market: evidence from quantile connectedness. Appl Econ.

[CR43] Pesaran HH, Shin Y (1998). Generalized impulse response analysis in linear multivariate models. Econ Lett.

[CR44] Phan DHB, Sharma SS, Tran VT (2018). Can economic policy uncertainty predict stock returns? Global evidence. J Int Financ Markets Inst Money.

[CR45] Politis D, Romano J (1994). The stationary bootstrap. J Am Stat Assoc.

[CR46] Ren B, Lucey B (2022). A clean, green haven? Examining the relationship between clean energy, clean and dirty cryptocurrencies. Energy Econ.

[CR47] Roh T-Y, Byun SJ, Xu Y (2020). Downside uncertainty shocks in the oil and gold markets. Int Rev Econ Financ.

[CR48] Rouatbi W, Demir E, Kizys R, Zaremba A (2021). Immunizing markets against the pandemic: COVID-19 vaccinations and stock volatility around the world. Int Rev Financ Anal.

[CR49] Saeed T, Bouri E, Alsulami H (2020). Extreme return connectedness and its determinants between clean/green and dirty energy green investments. Energy Econ.

[CR50] Salisu AA, Ogbonna AE (2022). The return volatility of cryptocurrencies during the COVID-19 pandemic: assessing the news effect. Glob Finance J.

[CR51] Sebastião H, Godinho P (2021). Forecasting and trading cryptocurrencies with machine learning under changing market conditions. Financ Innov.

[CR52] Tachibana M (2018). Safe-haven and hedge currencies for the US, UK, and Euro area stock markets: a copula-based approach. Glob Financ J.

[CR53] Umar Z, Gubareva M (2020). A time-frequency analysis of the impact of the Covid-19 induced panic on the volatility of currency and cryptocurrency markets. J Behav Exp Finance.

[CR54] Umar Z, Adekoya OB, Oliyide JA, Gubareva M (2021). Media sentiment and short stocks performance during a systemic crisis. Int Rev Financ Anal.

[CR55] Umar Z, Gubareva M, Naeem M, Akhter A (2021). Return and volatility transmission between oil price shocks and agricultural commodities. PLoS ONE.

[CR56] Umar Z, Gubareva M, Teplova T (2021). The impact of Covid-19 on commodity markets volatility: analyzing time-frequency relations between commodity prices and coronavirus panic levels. Resour Policy.

[CR57] Umar Z, Gubareva M, Teplova T, Tran DK (2022). Covid-19 impact on NFTs and major asset classes interrelations: insights from the wavelet coherence analysis. Finance Res Lett.

[CR58] Umar Z, Yousaf I, Gubareva M, Vo XV (2022). Spillover and risk transmission between the term structure of the US interest rates and Islamic equities. Pac Basin Finance J.

[CR59] Umar Z, Sayed A, Gubareva M, Vo XV (2022). Influence of unconventional monetary policy on agricultural commodities futures: network connectedness and dynamic spillovers of returns and volatility. Appl Econ.

[CR61] Vuuren G, de Jong R (2017). A comparison of risk aggregation estimates using copulas and Fleishman distributions. Appl Econ.

[CR62] Woebbeking F (2021). Cryptocurrency volatility markets. Digit Finance.

[CR63] Xia T, Ji Q, Zhang D, Han J (2019). Asymmetric and extreme influence of energy price changes on renewable energy stock performance. J Clean Prod.

[CR64] Xu M, Chen X, Kou G (2019). A systematic review of blockchain. Financ Innov.

[CR65] Xu Q, Zhang Y, Zhang Z (2021). Tail-risk spillovers in cryptocurrency markets. Finance Res Lett.

[CR66] Yang M-Y, Wu Z-G, Wu X (2022). An empirical study of risk diffusion in the cryptocurrency market based on the network analysis. Finance Res Lett.

[CR67] Yousaf I, Nekhili R, Gubareva M (2022). Linkages between DeFi assets and conventional currencies: evidence from the COVID-19 pandemic. Int Rev Financ Anal.

[CR68] Yousaf I, Qureshi S, Qureshi F, Gubareva M (2023). Connectedness of COVID vaccination with economic policy uncertainty, oil, bonds, and sectoral equity markets: evidence from the US. Ann Oper Res.

[CR69] Zhang J, He Q-Z (2021). Dynamic cross-market volatility spillover based on MSV model: evidence from Bitcoin, gold, crude oil, and stock markets. Complexity.

[CR70] Zhang Y, Wanga M, Xionga X, Zou G (2021). Volatility spillovers between stock, bond, oil, and gold with portfolio implications: evidence from China. Finance Res Lett.

[CR71] Cong LW, Li X, Tang K, Yang Y (2022) Crypto wash trading. National Bureau of Economic Research, Working Paper 30783. http://www.nber.org/papers/w30783

[CR72] FSB (2022) FSB statement on international regulation and supervision of crypto-asset activities, Financial Stability Board-FSB, https://www.fsb.org/wp-content/uploads/P110722.pdf

[CR73] Makarov I, Schoar A (2022) Cryptocurrencies and decentralized finance (DeFi). National Bureau of Economic Research-NBER, Working Paper 30oo6. http://www.nber.org/papers/w30006

[CR74] Ryvkin A (2019) Volatility products and their uses: an introduction to the VIX index and volatility instruments. Honors College Theses. 230. https://digitalcommons.pace.edu/honorscollege_theses/230

